# Tumor-associated macrophages in head and neck carcinoma: clinicopathological correlations and implications for immunotherapy

**DOI:** 10.1007/s00262-025-04282-y

**Published:** 2026-01-27

**Authors:** Diane Evrard, Aurélie Beaufrère, Clément Dumont, Séréna Louërat, Adrien Chaud, Alice Guyard, Samira Laouirem, Miguel Albuquerque, Annemilaï Tijeras-Raballand, Anne Couvelard, Valérie Paradis, Caroline Halimi, Éric Raymond, Muriel Hourseau, Sandrine Faivre

**Affiliations:** 1 Department of Otorhinolaryngology-Head and Neck Surgery, Bichat Hospital, AP-HP. Nord-Université Paris Cité, 46 Rue Henri Huchard, 75018 Paris, France; 2 INSERM UMR 1149, Centre de Recherche Sur L’Inflammation, Paris, France; 3 Department of Pathology, Beaujon Hospital, FHU MOSAIC, SiRIC InsiTu, AP-HP. Nord-Université Paris Cité, Clichy, France; 4 Medical Oncology Department, Saint-Louis Hospital, AP-HP. Nord-Université Paris Cité, Paris, France; 5 Department of Pathology, Bichat Hospital, AP-HP. Nord-Université Paris Cité, Paris, France; 6 AFR Oncology, 92012 Boulogne-Billancourt, France; 7 Medical Oncology Department, Saint-Joseph Hospital, Paris, France

**Keywords:** Head and neck squamous cell carcinoma, Tumor-associated macrophages, PD-1 inhibitors, Immunotherapy resistance, Perioperative immunotherapy, Tumor microenvironment

## Abstract

Tumor-associated macrophages (TAMs) and other PD-L1-expressing immune cells play a key role in head and neck squamous cell carcinoma (HNSCC). As PD-1 inhibitors have become standard therapy for recurrent/metastatic disease and are now used perioperatively in locally advanced resectable cases, understanding their impact on TAMs dynamics is critical. This study investigated the association between clinical features and TAMs prevalence in HNSCC, and their potential role in tumor progression and resistance to PD-1 blockade. Tumor samples from HNSCC patients were analyzed by simplex and multiplex immunohistochemistry, and fresh tumor slices were cultured ex vivo with PD-1 inhibitors. CD68 + and CD163 + macrophages and PD-L1 expression were quantified and correlated with clinical parameters. In a tissue microarray cohort of 96 patients, CD68 + and CD163 + macrophages were more abundant in oral and oropharyngeal tumors compared to laryngeal and hypopharyngeal carcinomas (*p* = 0.001 and *p* = 0.06, respectively). In 10% of cases, TAMs formed a barrier between tumor nests and immune infiltrates. Among nine patients with matched pre- and post-immunotherapy samples, TAMs density significantly increased post-treatment (*p* = 0.01 and *p* = 0.03), with CD163 + macrophages clustering at the tumor periphery in 78% of cases. Multiplex staining confirmed this spatial reorganization. Ex vivo exposure to PD-1 inhibitors reproduced the increase in TAMs. These results suggest that PD-1 blockade may foster M2-like macrophage accumulation, potentially contributing to immune evasion. This underscores the need for prospective studies and the development of macrophage-targeted strategies, particularly in the emerging context of perioperative immunotherapy in HNSCC.

**Supplementary Information:**

The online version contains supplementary material available at 10.1007/s00262-025-04282-y.

## Introduction

Head and neck squamous cell carcinomas (HNSCCs), encompassing malignancies of the oral cavity, pharynx, and larynx, represent a major global health burden, with 743,000 new cases and 362,000 deaths reported worldwide in 2020, primarily due to locoregional or distant progression [1]. The immune microenvironment of these tumors has become a major focus of investigation, particularly in the era of immunotherapy. In silico analyses, such as CIBERSORT, have revealed that monocytes and macrophages constitute the predominant immune cell populations infiltrating most solid tumors, including HNSCCs, where they account for 20–28% of tumor-infiltrating leukocytes [2].

Tumor-associated macrophages (TAMs), often classified along a phenotypic spectrum from pro-inflammatory, antitumoral M1 to immunosuppressive, pro-tumoral M2, play critical roles in modulating tumor behavior [3–6]. Immunohistochemistry using antibodies against CD68 allows the identification of all macrophages regardless of their phenotype, while CD163, a hemoglobin–scavenger receptor highly expressed by M2 macrophages, is used to further distinguish M1 from M2 subsets [7]. Notably, the abundance of TAMs, especially CD68 + and CD163 + subsets, has been associated with poor prognosis in HNSCC, as highlighted by recent meta-analyses [8].

While most studies have focused on TAMs in oral squamous cell carcinoma (OSCC), their role across the full spectrum of HNSCC sites remains less well characterized [9–29]. Despite heterogeneity in methodologies, there is consistent evidence linking high TAMs infiltration to tumor progression and therapy resistance. In particular, TAMs mediate multiple immunosuppressive mechanisms, including PD-L1 expression, IL-10 production, and recruitment of regulatory T cells, while also promoting angiogenesis and metastasis through various cytokines and matrix remodeling enzymes [30–34].

Importantly, TAMs themselves can express both PD-1 and PD-L1, potentially modulating antitumor immunity and influencing responses to PD-1 inhibitors [35, 36]. The clinical relevance of this interaction has grown considerably with the widespread use of PD-1 inhibitors in recurrent/metastatic HNSCC, and more recently, their approval as perioperative treatment for resectable, locally advanced disease—as evidenced by the positive results of trials such as KEYNOTE-689 and NIVOPOSTOP [37, 38].

In this evolving landscape, deciphering mechanisms of resistance to immune checkpoint inhibitors is an urgent priority. TAMs have emerged as potential contributors to immune escape, making them compelling targets for therapeutic modulation.

In this study, we aimed to (1) evaluate the association between clinical factors and TAMs frequency in HNSCC, and (2) investigate the role of TAMs in tumor progression and resistance to PD-1 blockade, using ex vivo tumor models and paired clinical samples.

## Materials and methods

### Patients and tissue specimens

Medical records, radiological data and histological specimens were collected from patients with HNSCC receiving treatment at the Department of Head and neck Surgery of Bichat hospital (AP-HP. Nord-Université Paris Cité). For all tumors, TNM classification and clinical staging followed International Union Against Cancer (UICC) guidelines.

For tissue microarrays (TMA) analysis, we included 100 patients with primary HNSCC treated with wide excision surgery from January 2009 to December 2011, with or without past history of HNSCCs treatment (radiotherapy or chemoradiotherapy) and neoadjuvant treatment (chemotherapy by paclitaxel, platinum and 5-fluorouracil). Histological samples restricted to HNSCCs biopsies were excluded. Among 100 tumor specimens, 96 containing tumoral and peritumoral tissue were evaluable.

For tissue slice culture experiments, fresh tumor samples were obtained from 11 patients referred for surgical HNSCC resection from January 2018 to June 2018. Four of the 11 tumor samples were not evaluable because necrosis was too extensive (*n* = 2) or tumor slices were not cultured with nivolumab (*n* = 2).

For 9 patients, histological specimens were collected before and after treatment by PD-1 inhibitor.

Overall survival (OS) was defined as the time from diagnosis to death from any cause, and disease-free survival (DFS) as the time from surgery to recurrence or death, whichever occurred first.

All patients provided written consent for research use of surgery and biopsy specimens. Tumor biopsies after immunotherapy at the time of recurrence were obtained by a head and neck surgeon from a primary tumor site or cutaneous nodule metastasis.

### Immunohistochemistry

Formalin-fixed paraffin-embedded tumor blocks were obtained from Bichat hospital pathology department files, and TMA sample was produced by extracting two to four punches of 2 mm from each surgical specimen, containing tumor and peritumoral tissue.

Immunohistochemistry (IHC) analyses were performed on each TMA: sections of 3–5 μm thickness were cut using a microtome. Sections were then placed onto Superfrost Plus adhesive slides and dried overnight at 37 °C or incubated for 1 to 2 h at 55 °C. IHC staining involved using a Roche Ventana Benchmark XT automated slide stainer (Ventana Medical Systems, Roche, France). The sections were incubated overnight with antibodies for CD68 (mouse monoclonal, 1:100, clone KP1, M0814, Dako Corp., Santa Barbara, CA, USA), CD163 (mouse monoclonal, 1:200, clone 10D6, NCL-CD163, Leica Biosystems, Nussloch, Germany), and PD-L1 (rabbit monoclonal, 1:400, 13,683, Cell Signaling Technology, Danvers, MA, USA) at 4 °C, then incubated with appropriate secondary antibodies.

For several tumor samples before and after immunotherapy, co-expression of CD68 (same antibody, dilution 1:500) and AE1/AE3 (mouse monoclonal, 1:20, M35150, Dako Corp., Santa Barbara, CA, USA) were analyzed.

### Precision-cut tumor slices

After surgical resection, a tumor sample measuring 0.5–1 cm was selected macroscopically from a viable, non-necrotic area of each tumor. Tumor samples were immediately immersed in Dulbecco’s Modified Eagle Medium (Gibco, 31,331–028) and prepared into slices in less than 1 hour. After agar embedding of the tumor sample, the tumor slices were prepared using a microtome equipped with a vibrating blade (thickness of the slices: 300 μm; speed of blade 0.2 mm/s; amplitude 3 mm; Vibratome VT1200S; Leica, Nanterre, France) under immersion in cold phosphate-buffered saline. Then, slices were carefully transferred to separate wells of a 24-well culture plate filled with cell culture medium consisting of Dulbecco’s Modified Eagle Medium supplemented with 10% fetal calf serum (PAN Biotech-GmbH, Aidenbach, Germany), 2 mM glutamine, and antibiotics (penicillin and streptomycin). Two slices in duplicate were cultured and analyzed for each condition. Nivolumab was purchased from Bristol-Myers Squibb and was used at a final concentration of 0.1, 0.5, 1 or 2 μM. These concentrations were selected based on previous in vitro and ex vivo studies demonstrating effective PD-1 blockade within a similar range without inducing tissue toxicity [39, 40]. The plates were incubated for 24 or 48 h in a chamber maintained at 37 °C and 5% CO2 atmosphere. The percentage of tumor necrosis was assessed from hematoxylin and eosin staining (HES) on the overall section by expert pathologists to ensure sufficient viability of tissue slides. The time point of 48 hours was selected on the basis of previous analyses [41, 42].

Each slice was fixed in 10% buffered formalin and embedded in paraffin. The paraffin-embedded tumor slices were used for histological (HES) and IHC analysis including CD68, CD163, PD-L1, and Ki67 (mouse monoclonal, 1:100, clone MIB-1, M7240, Dako Corp., Santa Barbara, CA, USA).

### Evaluation of immunoreactivity

Our analysis reports the results according to tumor components (i.e., tumor nest: area from which tumor cells originate, accumulate, or develop) and tumor stroma (microenvironment around the tumor cells).

For TMA and tumor biopsies after immunotherapy, immunoreactivity was semi-quantitatively evaluated by using an immunoreactive score. CD68 + and CD163 + macrophages were quantified by an expert pathologist specialized in head and neck cancers with masking to clinical data. As described by Lin et al., each TMA section was scored according to the number of macrophages in the tumor nest and in stroma as follows: GRADE 0, single/few positive cells; GRADE + 1, < 10% of the total number of cells were positive; GRADE + 2, > 10% and < 50% positive cells; GRADE + 3, > 50% positive cells [10, 18]. For each tumor, an average value from all individual TMA scores was calculated. For cut-off point analysis, the medians were used to classify tumors into high and low groups. PD-L1 expression was assessed for both tumor cells and intratumoral inflammatory cells in the tumor nest and stroma [43, 44]. For tumor cells, the proportion of PD-L1-positive tumor cells was estimated semi-quantitatively with a score of 0, 1, 2, or 3 (i.e., < 1%, > 1% but < 5%, > 5% but < 10%, or > 10% PD-L1–positive cells). PD-L1-labeled specimens were scored as positive at a threshold of 5% of all stained cells (score ≥ 2). Tumor-infiltrating immune cells were evaluated as positive or negative for PD-L1 staining [20, 43]. The PD-L1 score for patients with multiple TMAs was defined as the highest among the scores of the individual TMAs.

For cultured tumor slices, the frequency of CD68 + and CD163 + macrophages from each slide involved a quantitative method. The number of labeled macrophages was counted in 3 random high-power fields (HPFs, 20×). The average of these three counts was then calculated. Cell proliferation of tumor slices was analyzed according to the method of Donnadieu et al. [42]. The ratio of Ki67-positive cells (brown stained nuclei/total number of nuclei × 100) was calculated automatically for each tumor slice by using the free plugin ImmunoRatio in ImageJ. For each case, 5 random HPFs (20×) were analyzed. The average of all the results was calculated to determine the cell proliferation index.

### Multiplex immunofluorescence

For 8 tumor samples before and after immunotherapy, multiplex immunohistochemistry (mIHC) was realized. Formalin-fixed paraffin-embedded tissue sections of 5 μm were deparaffinized according to standard protocol followed by antigen retrieval. Tissue sections were stained for mIHC on a Leica Bond RX autostainer (Leica Biosystems) as previously described [45, 46]. Samples were evaluated using six-color panel including CD68, CD163, PD-L1, and pan-cytokeratin. Antigen–antibody binding was visualized with the TSA-Opal reagents (Akoya Biosciences). Antigen retrieval was performed between antibody detection to prevent cross-reactivity. Tissue slides were incubated with DAPI (#FP1490, Akoya Biosciences) as counterstain and coverslipped with ProLongTM Diamond Antifade Mountant (#P36965, Thermo Fisher Scientific). Control tissue samples were stained for each marker separately. Multispectral image analysis was performed using QuPath software (version 0.5.1), on the entire tissue area for each multiplexed image.

### Statistical analysis

The association between the CD68-positive and CD163-positive macrophages, PDL1 expression and clinicopathological parameters were analyzed by Chi-squared test. OS rate was estimated by the Kaplan–Meier method and compared by the log-rank test. To further quantify the prognostic impact of each marker, univariate Cox proportional hazards models were applied to estimate hazard ratios (HR) and 95% confidence intervals (CI). Multivariate analysis involved backward selection of variables (gender, age, smoking history, chronic alcohol intake, immunodeficiency, clinical stage, T stage, location, histological differentiation, p16 status, extracapsular invasion, vascular invasion, lymph vessel invasion, neoadjuvant treatment, recurrence, and the frequency of CD68 + and CD163 + macrophages) by the Aikake information criteria. All data were analyzed by using R studio version 1.1.423 (for Mac, R Foundation for Statistical Computing, Vienna, Austria) with the following packages: survival, lattice, xlsx. *P* < 0.05 was considered statistically significant.

## Results

### Clinical studies

#### Frequency and distribution of TAMs in resected HNSCC

We enrolled 100 patients in the clinical study. A total of 96 TMA samples had adequate tumor present for IHC assessment of CD68, CD163, PD-L1, and p16 (four samples did not reach adequate tumor cellularity). Among 96 patients with evaluable TMA samples, the median age was 60 years (range 31–88) and the male/female ratio was 3/1. Overall, 29 patients (30%) had already received radiation and/or chemotherapy, including 7 (7%) who had radiation and/or chemotherapy radiation and/or chemotherapy < 1 year before surgery. Primary tumors were located in the oral cavity (OSCC, *n *= 41), oropharynx (OPSCC, *n* = 37), larynx (LSCC, *n* = 11), or hypopharynx (HSCC, *n *= 7). Among the 37 OPSCC cases, 12 were p16-positive (32%). Patient characteristics are summarized in Table 1.

**Table 1:** Patient and tumor characteristics (*n* = 96 patients)

Gender	
Male	75 (78)
Female	21 (22)
Age, years, median (range)	60 (31–88)
Site of primary tumor	
Oral cavity	41 (43)
Oropharynx	37 (39)
Larynx	11 (11)
Hypopharynx	7 (7)
Smoking history	73 (76)
Chronic alcohol intake	52 (54)
Immunodeficiency ^a^	11 (11)
Neoadjuvant treatment (chemotherapy)	4 (4)
Past history of (chemo)radiotherapy	25 (26)
Clinical stage	
I	16 (17)
II	17 (18)
III	15 (16)
IV	48 (50)
T stage	
T1	20 (21)
T2	35 (36)
T3	21 (22)
T4	19 (20)
N stage	
N0	46 (48)
N +	47 (49)
Histological differentiation	
Well	49 (51)
Moderate	31 (32)
Poor	15 (16)
Median TMA sample analyzed per tumor	3
OS (years)	3.9 (0.1;9.1)
DFS (years)	3.5 (0.1;9.1)

TMA tumor microarray, OS overall survival, DFS disease-free survival

^a^Etiology of immunodeficiency included HIV and severe diabetes

**Fig. 1:**
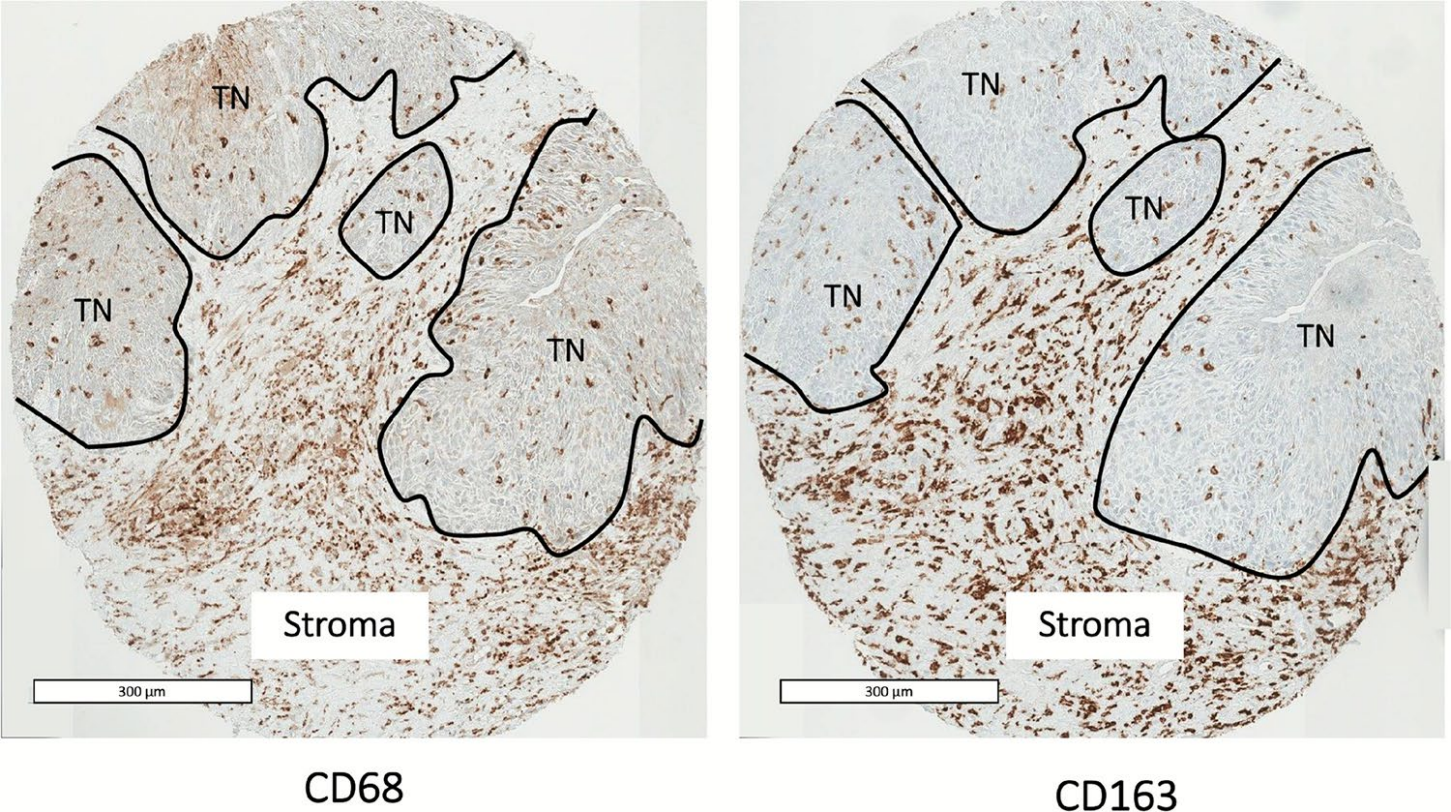
Immunohistochemistry of CD68-positive and CD163-positive macrophages found in the tumor nest (TN) and stroma. Scale bar = 300 μm

CD68 and CD163 markers were detected on the plasma membrane or in the cytoplasm of macrophages in both the tumor nest and stroma (Fig. 1). Use of a semi-quantitative score revealed 80% of tumor nests and 85% of stroma positive for CD68 + macrophages. For CD163 + macrophages, 75% of tumor nests and 82% of stroma were positive. Tumor cells also expressed CD68 and CD163 but with less intensity on the plasma membrane and in the cytoplasm (65% CD68 + and 7.3% CD163 + in tumor cells).

For tumor cells (TCs), PD-L1 expression (> 5%) was high in 32% of cases. For immune cells (ICs), 52% of cases were positive for PD-L1. The frequency of CD68 + and CD163 + macrophages was significantly associated with PD-L1 expression by TCs and ICs (Table S1).

**Fig. 2:**
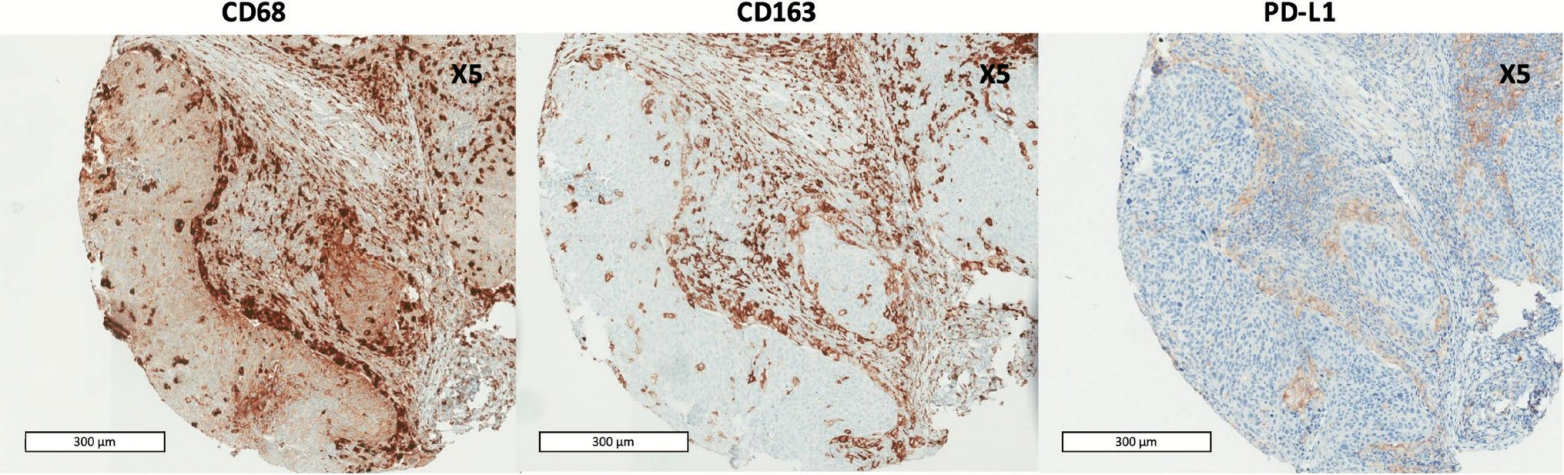
Immunohistochemistry of CD68 + , CD163 + , and PD-L1 + macrophages in the periphery of tumor nests. Scale bar = 300 μm

In 10 cases (10%), CD68 + CD163 + macrophages were observed at the periphery of the tumor nests (Fig. 2). For all these cases, TAMs with this distribution also appeared to express PD-L1.

#### Frequency of TAMs by primary tumor site and clinicopathologic features

CD68 + macrophages in the tumor nest were more frequent in OSCC and OPSCC than laryngeal and hypopharyngeal carcinomas (72% of OSCC and OPSCC vs 28% of LSCC and HSCC, *p* = 0.001) (Table 2). CD163 + macrophages in the tumor nest were also more frequent in OSCC and OPSCC than LSCC and HSCC, although not significantly (28% of OSCC and OPSCC vs 6% of LSCC and HSCC, *p* = 0.06) (Table 2).

**Table 2:** Univariate analysis of the association of clinical factors and CD68 + and CD163 + macrophage counts in the tumor nest and tumor stroma of HNSCC

		CD68 + macrophage count						CD163 + macrophage count					
		In tumor nest			In tumor stroma			In tumor nest			In tumor stroma		
Variable	No. of patients	Low	High	*p* value	Low	High	*p* value	Low	High	*p* value	Low	High	*p* value
All patients	96	35	61		38	58		73	23		43	53	
Tumor localization				**0.001**			0.20			0.06			0.45
OSCC/OPSCC	78	22	56		28	50		56	22		33	45	
LHSCC	18	13	5		10	8		17	1		10	8	
Gender				0.27			0.68			0.77			0.59
Female	21	5	16		7	14		17	4		11	10	
Male	75	30	45		31	44		41	34		32	43	
Age, years				0.25			**0.04**			0.15			0.65
≤ 50	14	3	11		2	12		8	6		5	9	
> 50	82	32	50		36	46		65	17		38	44	
Smoking history				0.50			0.62			1			1
No	21	6	15		7	14		16	5		9	12	
Yes	73	29	44		31	42		56	17		33	40	
Chronic alcohol intake				0.78			0.69			1			1
No	39	13	26		14	25		30	9		12	27	
Yes	52	20	32		22	30		41	11		16	36	
Prior therapy				0.67			0.64			1			0.82
No	67	23	44		25	42		51	16		29	38	
Yes	29	12	17		13	16		22	7		14	15	
T stage				**0.005**			** < 0.001**			**0.007**			** < 0.001**
T1-T2	55	13	42		13	42		36	19		16	39	
T3-T4	41	22	17		25	16		36	4		27	14	
N stage				0.61			1			1			0.61
N0	46	19	27		19	27		36	10		23	23	
N +	47	16	31		19	28		36	11		20	27	
Pathological grade				0.63			0.47			0.37			0.63
I	49	19	30		22	27		38	9		24	25	
II	31	9	22		10	21		20	10		12	19	
III	15	6	9		5	10		11	4		6	9	
Histological stage				0.52			0.13			0.40			0.35
Early	32	10	22		9	23		22	10		12	20	
Advanced	62	25	37		29	33		46	13		31	31	
Capsular rupture				0.90			0.63			0.89			0.72
No	72	27	45		27	45		54	18		31	41	
Yes	24	8	16		11	13		19	5		12	12	
Perineural invasion				0.32			0.37			1			1
No	69	28	41		30	39		52	17		30	39	
Yes	26	10	16		8	18		20	6		12	14	
Lymphovascular invasion				0.92			0.84			1			0.96
No	77	28	49		32	45		58	19		33	44	
Yes	17	7	10		6	11		13	4		8	9	
Follow-up				0.27			**0.01**			0.82			0.76
Live	43	16	27		13	30		33	10		20	23	
Death	44	18	26		24	20		34	10		20	24	
Lost to follow-up	9	1	8		1	8		6	3		3	6	
Recurrence				0.76			0.14			0.70			0.87
No	42	14	28		12	30		30	12		19	23	
Yes	38	14	24		18	20		30	8		18	20	
Lost to follow-up	16	7	9		8	8		13	3		6	10	

OSCC oral squamous cell carcinoma, OPSCC oropharyngeal squamous cell carcinoma, LHSCC laryngeal and hypopharyngeal squamous cell carcinoma, N0 no lymph node metastasis, N ( +*) node metastasis*

Bold values signify *p* < 0.05

The frequency of CD68 + and CD163 + macrophages in the tumor nest was significantly higher in early-stage T1-2 than late-stage T3-4 tumors (*p* = 0.005 and *p* = 0.007). Consistently, the frequency of CD68 + and CD163 + macrophages in tumor stroma was significantly higher in early-stage T1-2 than late-stage T3-4 tumors (*p* < 0.001 and *p* < 0.001) (Table 2). In OPSCC, the frequency of CD68 + and CD163 + macrophages in the tumor nest and stroma was not associated with p16 positivity (Table S2).

We performed a multivariate analysis of the association of specific variables (patients’ demographic characteristics, pre-existing conditions, lifestyle factors, clinical and histological tumor characteristics) and frequency of CD68 + and CD163 + macrophages. The frequency of CD68 + macrophages in the tumor nest remained high in OSCC and OPSCC primary sites (*p* = 0.01). Moreover, that of CD68 + and CD163 + macrophages in tumor stroma remained high in early T1-T2 stages (*p* = 0.04 and *p* = 0.04).

In an survival analysis of patients with stage T1-2 tumors (*n* = 55) that were most often enriched in CD163 + TAMs, OS was reduced but not significantly with CD163 + infiltrates in the tumor nest (HR = 1.56, 95% CI 0.66–3.64, log-rank *p* = 0.34, Fig. S1). There was no apparent association between CD68 + infiltrates and OS (HR = 1.05, 95% CI 0.39–2.84, log-rank *p* = 0.93, Fig. S1). For patients with stage T3-4 tumors (*n* = 41), OS was not associated with CD68 + and CD163 + infiltrates (Fig. S2).

When considering all patients (*n* = 96), OS tended to be lower in cases with low stromal PD-L1 expression or low p16 expression, though these differences were not statistically significant (PD-L1 in stroma: HR = 0.57, 95% CI 0.25–1.27, log-rank *p* = 0.16; Fig. S3; p16 expression: HR = 0.55, 95% CI 0.17–1.77, log-rank *p* = 0.31; Fig. S4).

#### Effect of PD-1 inhibitor on TAMs density

Histological specimens were available before and after treatment with a PD-1 inhibitor in 9 patients. All paired samples were analyzed using IHC and a subset of 8 paired samples was further analyzed with mIHC. Six patients (67%) received pembrolizumab and 3 (33%) nivolumab. Three patients (33%) received concomitant immunochemotherapy. Before treatment, 3 surgical specimens and 6 biopsies were available; after treatment, 2 surgical specimens and 7 biopsies were available. The median time from treatment initiation to biopsy was 6 months (range 1–16), and the median PD-1 inhibitor treatment was 5 cycles (range 2–11). Patient characteristics are summarized in Table 3.

**Table 3:** Characteristics of patients with pre- and post-immunotherapy paired biopsies

Patient	Gender	Age	Site of primary tumor	Pattern of relapse	Treatment	Delay between IT initiation and biopsy (months)		On-treatment change in CD68 staining	On-treatment change in CD163 staining
1	M	66	Oral cavity	LRR	Nivolumab	6	PR (− 85%)	Increase	Increase
2	M	64	Larynx	Lung Me	Nivolumab	7	SD (lung) then Radiological LR progression	Increase	Increase
3	M	72	Oral cavity	LRR	Nivolumab	7	PR (− 75%) then progression (+ 66%)	Decrease	Decrease
4	F	65	Oropharynx	LRR	Pembrolizumab	2	Early clinical progression^a^	Increase	Increase
5	F	68	Larynx	LRR + Lung Me	Pembrolizumab	16	SD (10 months) then Radiological progression (+ 38%)	Increase	Stable
6	M	55	Oral cavity	LRR	Pembrolizumab	9	Radiological progression (+ 50%)	Increase	Increase
7	M	53	Hypopharynx	LRR	Pembrolizumab, Carboplatin	5	Radiological progression (+ 42%)	Increase	Increase
8	M	53	Oropharynx	LRR	Pembrolizumab, Carboplatin, 5-FU	2	Radiological progression (+ 29%)	Stable	Stable
9	M	72	Hypopharynx	LRR	Pembrolizumab, Carboplatin, 5-FU	1	Early clinical progression^a^	Increase	Increase

CR complete response, F female, IT immunotherapy, LR locoregional, LRR locoregional recurrence, M male, Me metastasis, PR partial response, SD stable disease

^a^Early clinical progression did not allow for performing imaging

**Fig. 3:**
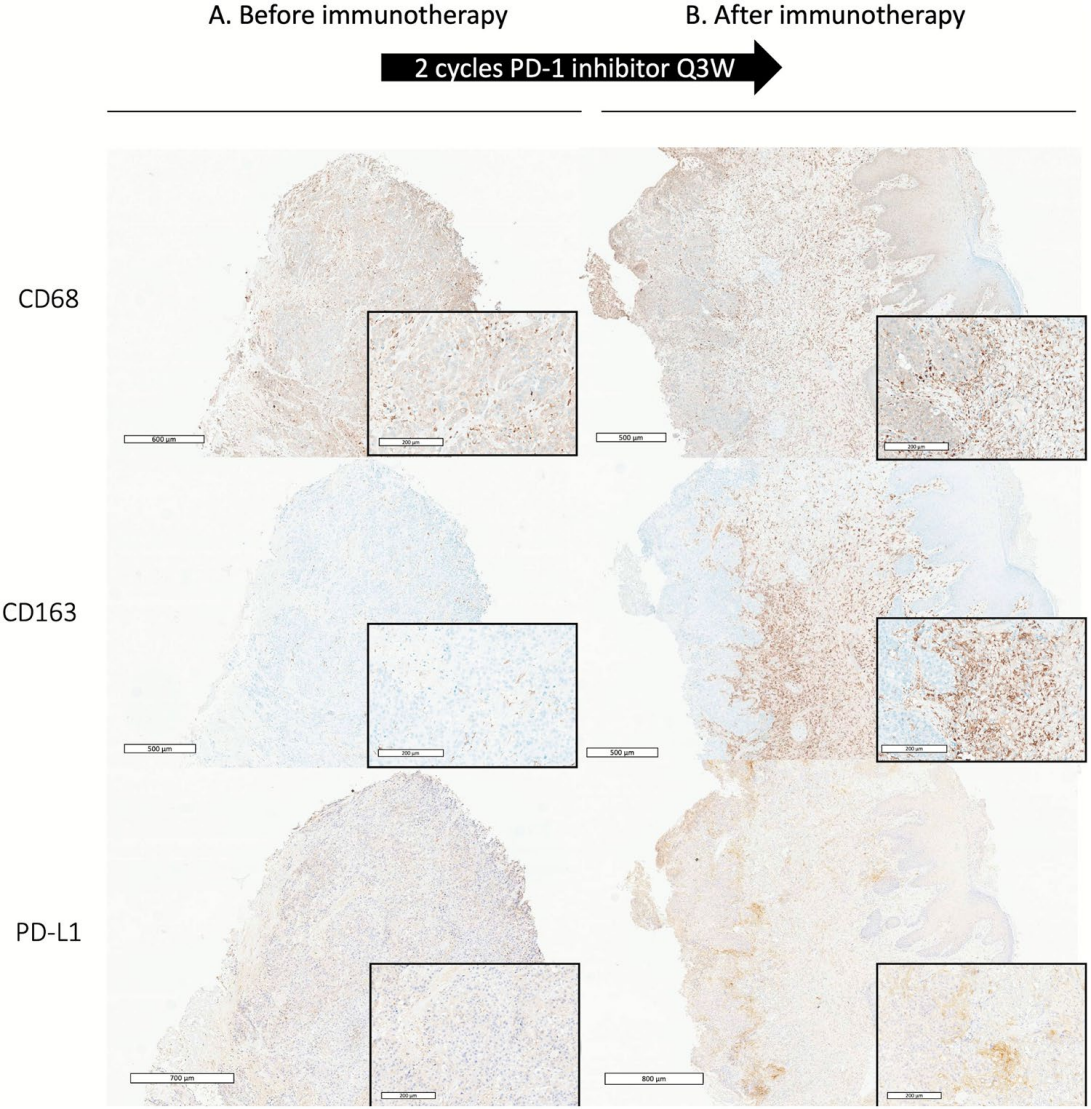
Representative immunohistochemistry examples of the frequency of CD68 + and CD163 + macrophages and PD-L1 staining before (A) and after (B) 2 cycles of PD-1 inhibitor treatment, resulting in early progression. Scale bar = 500–800 μm. The inserts (scale bar = 200 μm) show higher magnification images

After immunotherapy, the semi-quantitative score was increased significantly for CD68 + macrophages (3.6 vs 2.4, *p* = 0.01) and CD163 + macrophages (3.6 vs 2.3, p = 0.03) (Fig. 3 and S5).

**Fig. 4:**
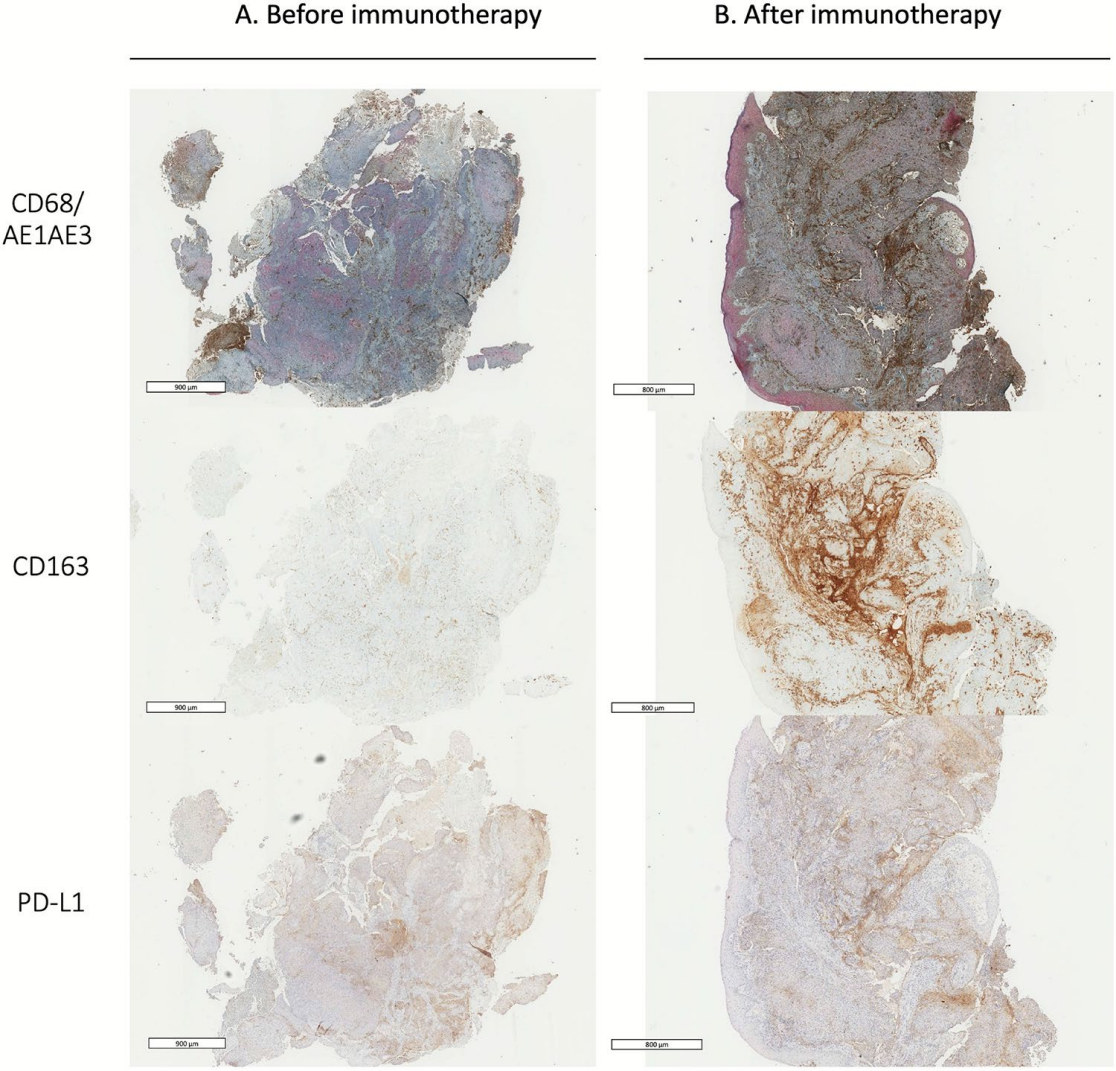
Representative immunohistochemistry examples of the distribution of CD68 + and CD163 + macrophages and PD-L1 staining before (A) and after (B) 11 cycles of PD-1 inhibitor treatment. Co-expression of CD68 (in brown) and epithelial marker AE1/AE3 (in red) was analyzed. Scale bar = 800–900 μm

After immunotherapy, the frequency of CD163 + macrophages increased and such macrophages were predominant at the periphery of the tumor nest and almost exclusively in the stroma (Fig. 4) in 7 cases (78%). Among those 7 cases, only one case had this distribution before immunotherapy.

**Fig. 5:**
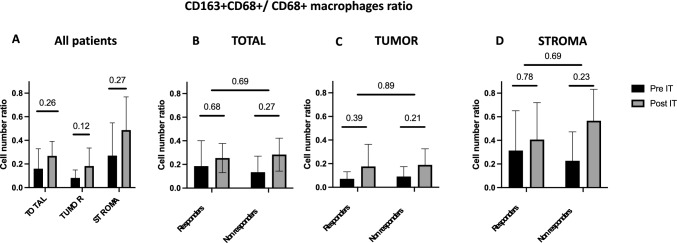
Effect of PD-1 inhibitor on CD163 + CD68 + / CD68 + macrophages ratio in all patients (*n* = 8), analyzed globally, in the tumor nest, and in the stroma (A). Comparison of CD163 + CD68 + / CD68 + macrophages ratio between responders (*n* = 4) and non-responders (n = 4) in total (B), in the tumor nest (C) and in the stroma (D). *IT, immunotherapy*

**Fig. 6:**
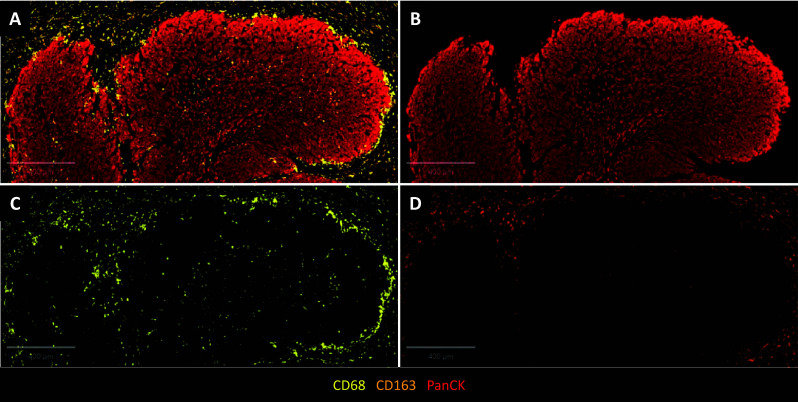
Representative multiplex immunohistochemistry highlighting the distribution of CD68 + (yellow) and CD163 + (orange) macrophages (A) at the periphery of tumor nest stained with panCK antibody (B, red) for a patient after 5 cycles of PD-1 inhibitor treatment. CD163 + remains at the periphery of tumor nest (D) while CD68 + macrophages were located both at periphery and also inside tumor nest (C). Scale bar = 400 μm

The increase in CD68 + /CD163 + macrophages and TAMs spatial distribution at the periphery of tumor nest after immunotherapy were confirmed on mIHC analyses (not significant). No correlation between responders (*n* = 4) and non-responders (*n* = 4) was found (Figs. 5 and 6).

### Ex vivo studies

**Fig. 7:**
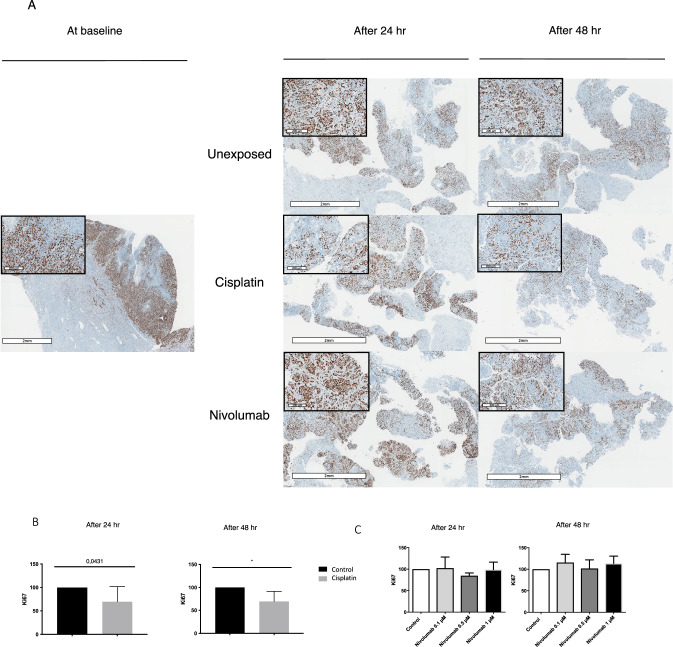
Representative immunohistochemistry examples of Ki67 at baseline, after 24 h and 48 h (A) in an unexposed control, in a case exposed to cisplatin and in a case exposed to nivolumab. Scale bar = 2 mm. The inserts (scale bar = 200–300 μm) show higher magnification images. (B) Effect of cisplatin on cell proliferation index (% of Ki67-positive cells) after 24 h (n = 7) and 48 h (n = 6) of exposure (p = 0.0431 and p = 0.0147). (C) Effect of nivolumab on cell proliferation index (% of Ki67-positive cells) after 24 h (n = 7) and 48 h (*n* = 6) of exposure

**Fig. 8:**
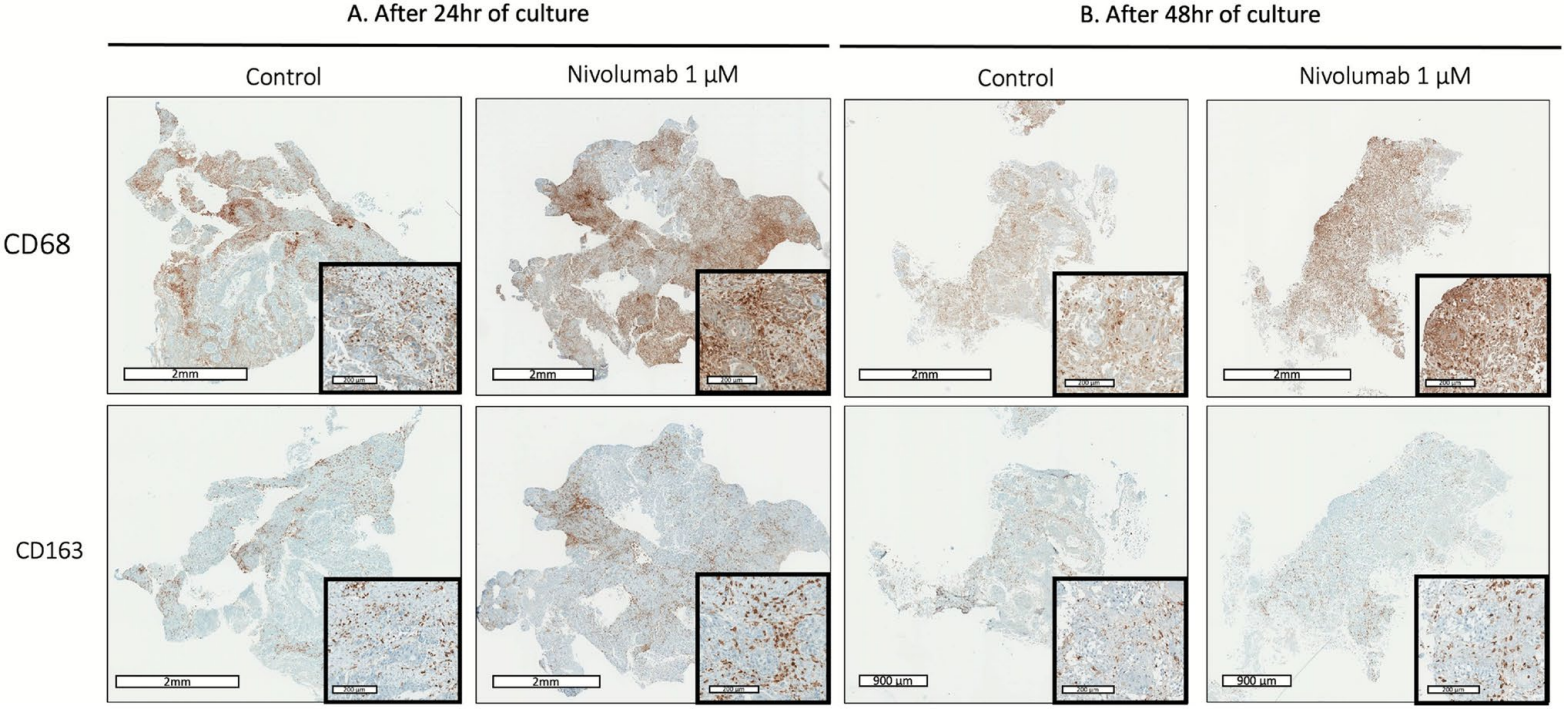
Representative immunohistochemistry examples of the frequency of CD163 + macrophages after 24 h (A) and 48 h (B) of exposure to nivolumab. Scale bar = 0.9–2 mm. The inserts (scale bar = 200 μm) show higher magnification images

To further explore the effect of PD-1 inhibition on frequency of TAMs in HNSCC, we used ex vivo culture of fresh tumor slices from 7 surgical specimens from HNSCC patients. We maintained tumor slices for 48 h in culture with nivolumab (PD-1 inhibitor) at different pharmacological concentrations, cisplatin (cytotoxic chemotherapy), or no drug (control). On IHC, we compared the cell proliferation index under each condition by using Ki67 staining and the frequency of TAMs by using CD68 and CD163 staining. The cell proliferation index (% of Ki67-positive cells) significantly decreased with cisplatin as compared with the control at 24 and 48 h of culture (*p* = 0.0431 and *p* = 0.0147) (Fig. 7). Nivolumab had no effect on the cell proliferation index (Fig. 7). CD68 + and CD163 + staining was increased although not significantly on tumor slices treated with nivolumab at 24 and 48 h (Fig. 8). The mean CD163 + macrophage count in three random high-power fields confirmed a dose-dependent but non-significant increase. At 24 h, as compared with the control (count: 100), the CD163 + macrophage count was 112 with 0.1 μM nivolumab, 124 with 0.5 μM, and 170 with 1 μM. At 48 h, the CD163 + macrophage count was 94, 117, and 123 for similar concentrations (Fig. S6).

## Discussion

In this study, we used clinically annotated histological specimens from patients with HNSCC to evaluate the distribution and frequency of TAMs and to explore their role in tumor progression and potential resistance to immunotherapy.

While only a few studies have investigated TAMs distribution across all HNSCC sites [27, 28], most existing literature focuses primarily on OSCC [9–14]. Our results show that the density of TAMs within the tumor nest is higher in OSCC and OPSCC than in laryngeal and hypopharyngeal carcinomas. This site-specific difference is consistent with the literature and highlights the importance of considering tumor location when evaluating the prognostic and therapeutic implications of TAMs in HNSCC [8, 47]. One plausible explanation for the higher TAMs frequency in OSCC and OPSCC is the increased exposure of their mucosae to environmental factors and oral microbiota, in contrast to more protected regions such as the larynx or hypopharynx. This hypothesis is supported by Hu et al., who reported more CD68 + macrophages in anterior oral subsites like the gingiva and buccal mucosa than in the tongue [9]. However, contrary findings by Seminerio et al. showed a higher density of CD68 + macrophages in LSCC compared to HSCC and OPSCC [27]. If our findings are confirmed in larger cohorts, the impact of primary anatomic sites (OSCC and OPSCC versus LSCC and HSCC) might be of potential relevance for TAMs distribution and immunotherapy outcomes.

We also observed a peritumoral distribution of TAMs in 10% of our TMA samples. This pattern has previously been described as forming an “immune barrier,” which may contribute to immune evasion, as shown by Lyford-Pike et al. in HNSCC [48] and more recently in hepatocellular carcinoma, where peritumoral macrophage accumulation was associated with resistance to immunotherapy [49]. In our cohort, this distribution did not correlate with clinical outcomes, but the subgroup was small, and the TMA format limited our ability to sample intratumoral heterogeneity.

Interestingly, we found that TAMs frequency was significantly higher in early-stage (T1-T2) tumors than in advanced-stage (T3-T4) tumors, in both tumor nests and stromal compartments. The apparent reduction in TAMs density in advanced tumors may be explained by several factors, including hypoxia-induced functional exhaustion or redistribution of TAMs, altered chemokine gradients, and the formation of immunosuppressive niches that limit further macrophage recruitment or retention [50]. Moreover, advanced tumors often display necrosis and architectural remodeling that can affect immune cell localization and detection. The clinical significance of this pattern remains unclear, and further studies integrating phenotypic and spatial analyses are warranted. Moreover, a high TAMs count in early-stage tumors was associated with reduced OS, suggesting a potential prognostic role in early disease. These findings are in line with several previous studies reporting an association between high TAMs density and poor prognosis [7, 9, 11, 12, 14, 18].

Another key objective of this study was to assess the temporal dynamics of macrophages in response to immunotherapy. In both our ex vivo and clinical models, we observed an increase in CD163 + TAMs following treatment with PD-1 inhibitors. While this increase was statistically significant using IHC, this was not the case with mIHC and tumor slice culture model, likely due to smaller sample size. At this stage, the observed increase in TAMs after PD-1 inhibitors should be considered exploratory and hypothesis-generating, as no significant association with treatment response was identified. These observations align with growing evidence that TAMs populations in the tumor microenvironment  are modulated in the context of immune‑checkpoint blockade [51]. Multiple studies have reported that CD163⁺ macrophages are often enriched in the tumor microenvironment  following immunotherapy, and their presence has been linked to enhanced immunosuppression and reduced responsiveness to immune checkpoint inhibitors across various solid tumors. For example, Lo Russo et al. demonstrated that anti-PD-1 therapy increased infiltration of M2-like macrophages in lung cancer models and in patients with hyperprogressive disease [52]. In HNSCC, neoadjuvant anti-PD-1 trials have shown increased macrophage infiltration in post-treatment specimens, particularly in non-responders [53–56]. Ju et al. notably reported one case of hyperprogression associated with a marked increase in CD163 + TAMs [56] (Table S3). These clinical data appear to contradict certain preclinical models suggesting that PD-1/PD-L1 blockade skews macrophage polarization toward an antitumor M1 phenotype [51, 57]. However, it is important to emphasize that the relationship between increased TAMs levels and resistance to immunotherapy is not straightforward. For instance, recent spatial transcriptomics studies have shown that the localization of TAMs within the tumor microenvironment  has a greater impact on responses to immune checkpoint blockade than their overall abundance [58]. Additionally, the plasticity of TAMs under therapeutic pressure can lead to functional remodeling that, in some contexts, enhances antitumor immunity [59, 60]. Our results therefore support the significance of the temporal dynamics of CD163⁺ macrophages during immunotherapy, without establishing a direct or exclusive link to resistance. The apparent expansion of M2-like macrophages after PD-1 inhibition underscores the need for mechanistic studies and the development of TAMs-targeted strategies, possibly in combination with immune checkpoint inhibitors. These findings are particularly relevant in light of recent approval of perioperative immunotherapy for resectable, locally advanced HNSCC, as exemplified by the KEYNOTE-689 trial [38].

Moreover, improved phenotypic characterization of TAMs could enhance our understanding of their functional roles. Lo Russo et al. described “complete immunophenotype” clusters of epithelioid macrophages co-expressing CD163, PD-L1, and CD33 in all pretreatment tumors from patients with hyperprogressive disease [52]. Similarly, Donadon et al. observed that larger macrophage morphology in colorectal liver metastases was associated with poor prognosis, suggesting that both phenotypic and morphological features of TAMs may hold prognostic value [61]. Larger macrophages were more frequent in colorectal liver metastasis than in the liver controls. Moreover, larger macrophages were more abundant in patients with worse prognosis than those with good prognosis, which suggests that TAMs morphology could be a critical feature associated with distinct clinical outcomes.

We selected two immunohistochemical markers, CD68, as a pan-macrophage marker, and CD163, to identify M2-like macrophages, which could not entirely characterize the plasticity of TAMs. Although the M1/M2 classification, originally developed for in vitro monocyte-derived macrophages, remains widely used, it fails to encompass the full spectrum of macrophage diversity, particularly within the tumor microenvironment  [62–64]. Functional heterogeneity extends far beyond these markers, underscoring the need for future studies employing single-cell or multiplexed analytical approaches to achieve a more comprehensive characterization. Moreover, subgroup analyses, particularly those involving survival or peritumoral TAMs distribution, are limited by small sample sizes and by the intrinsic heterogeneity of HNSCC across anatomical subsites. Pooling all cases together may mask site-specific immune features, and the relatively low number of tumors in certain subsites (e.g., hypopharynx and larynx) further limits the robustness of these observations, which should therefore be interpreted with caution.

In summary, our findings indicate that TAMs frequency in HNSCC varies according to tumor location and may tend to increase following PD-1 inhibitor treatment. Although these observations suggest a potential involvement of TAMs in resistance to immunotherapy, the subgroup analyses were limited by sample size and should therefore be interpreted with caution. To address these limitations, we are currently launching a prospective translational study (Eudra-CT: 2023-A00841-44), which will integrate TAMs profiling with oral microbiota analysis in patients with HNSCC receiving immunotherapy.

## Supplementary Information

Below is the link to the electronic supplementary material.

Supplementary file1 (DOCX 4376 KB)

## Data Availability

The datasets generated during and/or analysed during the current study are available from the corresponding author on reasonable request.

## Abbreviations

CCL2: Chemokine ligand 2
CTL: Cytotoxic T lymphocyte
DFS: Disease-free survival
HSCC: Hypopharyngeal squamous cell carcinomas
HNSCCs: Head and neck squamous cell carcinomas
ICs: Immune cells
IHC: Immunohistochemistry
LSCC: Laryngeal squamous cell carcinomas
mIHC: Multiplex immunohistochemistry
OPSCC: Oropharyngeal squamous cell carcinomas
OS: Overall survival
OSCC: Oral squamous cell carcinomas
PD-L1: Programmed death ligand 1
TAMs: Tumor-associated macrophages
TCs: Tumor cells
TMA: Tissue microarrays
UICC: International union against cancer
