# Supplementary material for: Tumor-associated macrophages in head and neck carcinoma: clinicopathological correlations and implications for immunotherapy
Source: Cancer Immunol Immunother. 2026 Jan 27;75(2):51. doi: 10.1007/s00262-025-04282-y (PMC12847608; doi:10.1007/s00262-025-04282-y)
Supplement: Supplementary file 1 — Supplementary file1 (DOCX 4376 KB) [file 262_2025_4282_MOESM1_ESM.docx]

**Tumor-associated macrophages in head and neck carcinoma: clinicopathological correlations and implications for immunotherapy**

Diane Evrard ^1,2,*^, Aurélie Beaufrère ^2,3^, Clément Dumont ^4^, Adrien Chaud ^1^, Séréna Louërat ^1^, Alice Guyard ^5^, Samira Laouirem ^2^, Miguel Albuquerque ^3^, Annemilaï Tijeras-Raballand ^6^, Anne Couvelard ^2,5^, Valérie Paradis ^2,3^, Caroline Halimi ^1^, Éric Raymond ^7^, Muriel Hourseau ^5^, Sandrine Faivre ^2,4^

1 Department of Otorhinolaryngology, Bichat Hospital, AP-HP. Nord-Université Paris Cité, Paris, France;

2 Centre de Recherche sur l'Inflammation, INSERM UMR 1149, Paris, France

3 Department of Pathology, Beaujon Hospital, AP-HP. Nord-Université Paris Cité, FHU MOSAIC, Clichy, France

4 Medical Oncology Department, Saint-Louis Hospital, AP-HP. Nord-Université Paris Cité, Paris, France;

5 Department of Pathology, Bichat Hospital, AP-HP. Nord-Université Paris Cité, Paris, France

6 AFR Oncology, 92012 Boulogne-Billancourt, France

7 Medical Oncology Department, Saint-Joseph Hospital, Paris, France;

* Corresponding author : [diane.evrard@aphp.fr](mailto:evrard.diane@gmail.com)

**Corresponding author:**

Name : Diane Evrard

Address : Department of Otolaryngology-Head & Neck Surgery, Bichat Claude-Bernard Hospital, 46 Rue Henri Huchard, 75018 Paris, France

E-mail address: [diane.evrard@aphp.fr](mailto:diane.evrard@aphp.fr)

**Journal :** Cancer Immunology, Immunotherapy

**Supplementary Fig. 1** Kaplan–Meier overall survival curves for T1 and T2 head and neck squamous cell carcinoma according to the frequency of CD68⁺ and CD163⁺ macrophages in the tumor nest (blue line for low frequency and red line for high frequency) (n = 55).

No significant difference in overall survival was observed according to macrophage infiltration (CD68: HR = 1.05, 95% CI 0.39–2.84, log-rank p = 0.93; CD163: HR = 1.56, 95% CI 0.66–3.64, log-rank p = 0.34).

**Supplementary Fig. 2** Kaplan–Meier overall survival curves for T3 and T4 head and neck squamous cell carcinoma according to the frequency of CD68⁺ and CD163⁺ macrophages in the tumor nest (blue line for low frequency and red line for high frequency) (n = 41).

No significant difference in overall survival was observed according to macrophage infiltration (CD68: HR = 1.09, 95% CI 0.47–2.56, log-rank p = 0.84; CD163: HR = 0.37, 95% CI 0.05–2.73, log-rank p = 0.31).

**Supplementary Fig. 3** Kaplan–Meier overall survival curves for head and neck squamous cell carcinoma according to the PD-L1 expression in the tumor nest and in the stroma (blue line for low frequency and red line for high frequency) (n = 96).

No significant difference in overall survival was observed according to PD-L1 expression (Tumor nest : HR = 0.86, 95% CI 0.46–1.63, log-rank p = 0.65; Stroma : HR = 0.57, 95% CI 0.25–1.27, log-rank p = 0.16).

**Supplementary Fig. 4** Kaplan–Meier overall survival curves for head and neck squamous cell carcinoma according to the p16 expression (blue line for low frequency and red line for high frequency) (n = 96).

No significant difference in overall survival was observed according to p16 expression (HR = 0.55, 95% CI 0.17–1.77, log-rank p = 0.31).

**Supplementary Fig. 5** Images of clinical examination before (A) and after (B) 2 cycles of PD-1 inhibitor treatment, resulting in early progression.

**
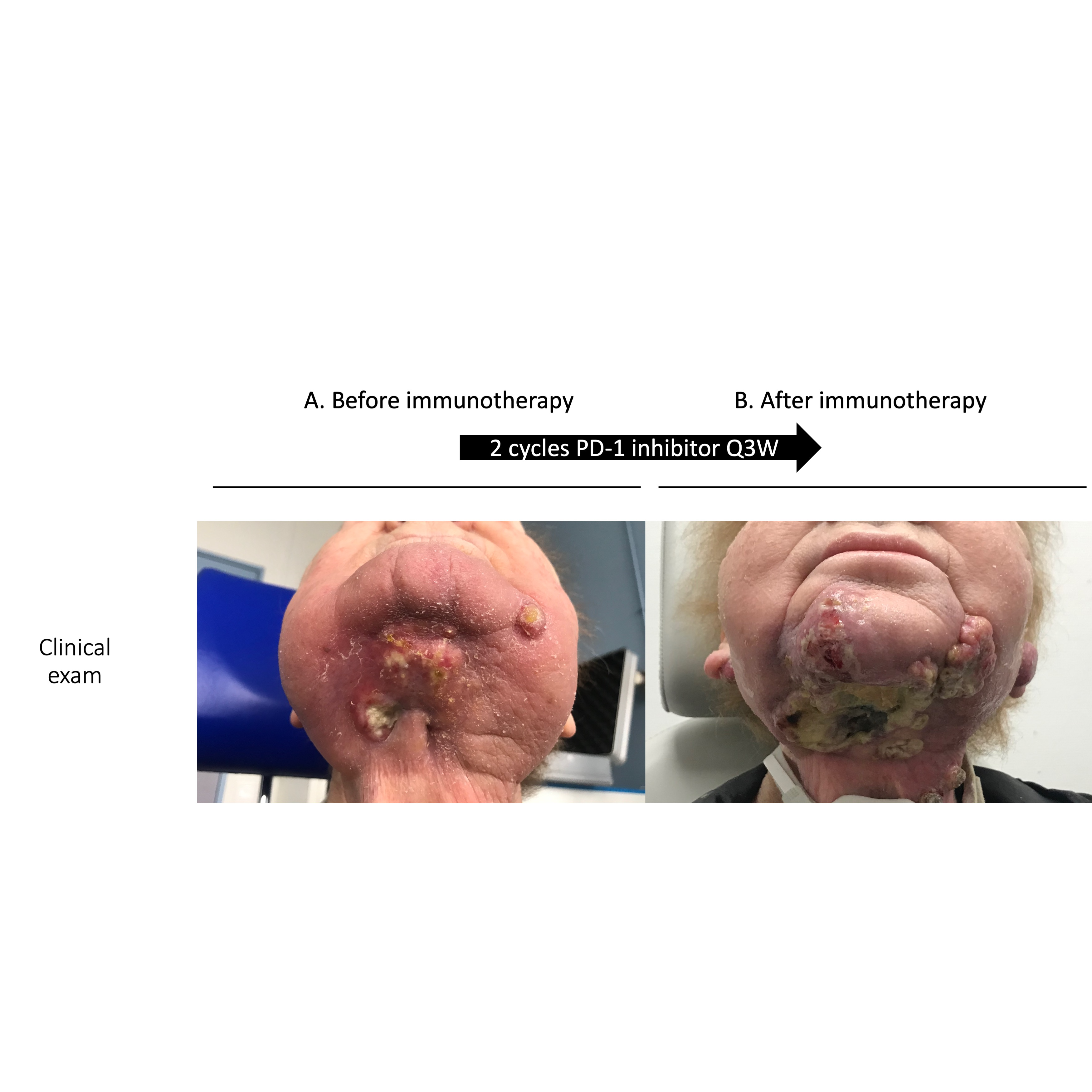
**

**Supplementary Fig. 6 Effect of nivolumab on frequency of macrophages CD163+ after 24 hours (n = 7) and 48 hours (n = 6) of exposure (p=0.3576 and p= 0.3329).**

**Supplementary Table 1.** **Univariate analysis of the association of CD68+ and CD163+ macrophage counts in tumor nests and tumor stroma and programmed cell death ligand 1 (PD-L1) expression on tumor cells and immune cells.**

|  |  |  | **PD-L1 expression** | | | | | | |
| --- | --- | --- | --- | --- | --- | --- | --- | --- | --- |
|  |  |  |  | **Tumor cells** | | | **Immune cells** | | |
|  |  |  | **n of patients** | **Low** | **High** | **p value** | **Negative** | **Positive** | **p value** |
|  |  |  |  |  |  | **0.008** |  |  | **<0.001** |
| CD68 | Tumor nest | Low | 35 | 30 | 5 |  | 26 | 9 |  |
|  |  | High | 61 | 35 | 26 |  | 20 | 41 |  |
|  |  |  |  |  |  | **0.01** |  |  | **<0.001** |
|  | Tumor stroma | Low | 38 | 32 | 6 |  | 29 | 9 |  |
|  |  | High | 50 | 31 | 19 |  | 17 | 41 |  |
|  |  |  |  |  |  | **0.002** |  |  | 0.47 |
| CD163 | Tumor nest | Low | 73 | 56 | 17 |  | 37 | 36 |  |
|  |  | High | 23 | 9 | 14 |  | 9 | 14 |  |
|  |  |  |  |  |  | **0.001** |  |  | **0.005** |
|  | Tumor stroma | Low | 43 | 37 | 6 |  | 28 | 15 |  |
|  |  | High | 53 | 28 | 25 |  | 18 | 35 |  |

**Supplementary Table 2 : Univariate analysis of the association of p16 status and CD68+ and CD163+ macrophage count in tumor nest and tumor stroma of oropharyngeal squamous cell carcinoma (n=37).**

|  | **CD68+ macrophages counts** | | | | | | | **CD163+ macrophages counts** | | | | | |
| --- | --- | --- | --- | --- | --- | --- | --- | --- | --- | --- | --- | --- | --- |
|  |  | **In tumor nest** | | | **In tumor stroma** | | | **In tumor nest** | | | **In tumor stroma** | | |
| **Variable** | **No.** | **Low** | **High** | **p value** | **Low** | **High** | **p value** | **Low** | **High** | **p value** | **Low** | **High** | **p value** |
| **P16** |  |  |  | 1 |  |  | 1 |  |  | 0.43 |  |  | 0.49 |
| Negative | 25 | 7 | 18 |  | 7 | 18 |  | 20 | 5 |  | 8 | 17 |  |
| Positive | 12 | 3 | 9 |  | 3 | 9 |  | 8 | 4 |  | 6 | 6 |  |

**Supplementary Table 3 :Analyses of tumor-associated macrophages in clinical trials with neoadjuvant immunotherapy for head and neck squamous cell carcinoma**

| **Study** | **Characteristics of patients** | **Treatment** | **Analyses of TAMs** | |
| --- | --- | --- | --- | --- |
|  |  |  | **Patients with response (R)/no response (NR)** | **Pre- and post-IT** |
| **Wise-Draper et al.** | 92 HNSCC (stage III or IV: T3 or T4 or ≥N2) and HPV- | 1 dose of pembrolizumab 7-21 days before surgery |  | ⬈ Gene expression signature of macrophages after IT |
| **Uppaluri et al.** | 36 HNSCC (stage III or IVb) and HPV- | 1 dose of pembrolizumab 7-21 days before surgery | More M1 in R (significant)  More M2 in R (not significant) | ⬈ M1 and M2 post IT for NR |
| **Ju et al.** | 20 OSCC  (stage III or IVa) | PD-1 inhibitor camrelizumab + VEGFR2 inhibitor |  | ⬈ CD68+/CD163+  ⬈ CD68+/CD163+ for NR  Hyperprogression:  ⬈ CD163+ |
| **Leidner et al.** | 21 HNSCC HPV+ (stages I–III) or HPV- (stages III– IVa) | 1 dose of nivolumab |  | ⬈ frequency of macrophages after IT |

*HNSCC head and neck squamous cell carcinoma, HPV human papillomavirus, IT immunotherapy, OSCC oral squamous cell carcinoma, TAMs tumor-associated macrophages VEGFR2 vascular endothelial growth factor receptor 2*
